# Impaired fasting glucose and elevated 180-minute OGTT values predict metformin failure in gestational diabetes mellitus: a retrospective cohort study

**DOI:** 10.1007/s00404-026-08528-7

**Published:** 2026-07-21

**Authors:** Sivan Farladansky-Gershnabel, May Weinberg, Reut Baram, Shani Saffer, Ilil Mishani-Sagi, Or Eliner, Tal Biron-Shental, Dorit Ravid

**Affiliations:** 1 Department of Obstetrics and Gynecology, Meir Medical Center, Kfar Saba, Israel; 2 Gray Faculty of Medical & Health Sciences, Tel Aviv University, Tel Aviv, Israel

**Keywords:** Gestational diabetes mellitus, Metformin, Insulin therapy, Oral glucose tolerance test, Impaired fasting glucose, Metabolic phenotype

## Abstract

**Purpose:**

To evaluate whether oral glucose tolerance test (OGTT)-derived metabolic abnormalities and maternal characteristics predict metformin failure in women with gestational diabetes mellitus (GDM).

**Methods:**

This retrospective cohort study included women with singleton pregnancies and gestational diabetes mellitus treated with metformin at a tertiary center (2018–2023). Gestational diabetes mellitus was diagnosed using the American Diabetes Association (ADA) two-step approach, consisting of an initial 50-g glucose challenge screening test followed, when abnormal, by a diagnostic 100-g, 3-h oral glucose tolerance test performed under fasting conditions and interpreted according to Carpenter-Coustan criteria (fasting ≥ 95 mg/dL, 1-h ≥ 180 mg/dL, 2-h ≥ 155 mg/dL, and 3-h ≥ 140 mg/dL). Routine screening was generally performed between 24 and 28 weeks’ gestation, with earlier testing in high-risk women when clinically indicated. Of 431 women who initiated metformin, 6 discontinued due to adverse effects, leaving 425 for analysis. Impaired fasting glucose (IFG) was defined as fasting OGTT ≥ 95 mg/dL. The primary outcome was metformin failure, defined as subsequent insulin initiation. Multivariable logistic regression included prespecified OGTT-derived phenotypes rather than simultaneous inclusion of all continuous OGTT variables in order to reduce multicollinearity.

**Results:**

Among 425 women, 114 (26.8%) required insulin. These women had higher glucose values at all OGTT time points and a higher prevalence of IFG (41.2% vs 26.4%, p = 0.005). An abnormal 180-min value was also more frequent (21.1% vs 5.1%, p < 0.001). The number of abnormal OGTT values did not differ significantly between groups and was not independently associated with metformin failure. In multivariable analysis, IFG (aOR 1.96, 95% CI 1.25–3.07) and an abnormal 180-min OGTT value (aOR 4.92, 95% CI 2.50–9.66) remained independently associated with metformin failure.

**Conclusion:**

IFG and an elevated 180-min OGTT value may identify women with GDM at higher risk for treatment escalation. These findings should not be interpreted as direct indications for insulin initiation but rather as potential markers of increased risk for metformin failure requiring closer glycemic surveillance.

## What does this study adds to the clinical work?

**Table:** 

Among women with GDM treated initially with metformin, impaired fasting glucose and an abnormal 180-minute OGTT value independently identified a higher likelihood of requiring insulin. These routinely available OGTT parameters may support closer glycemic surveillance and more individualized treatment escalation, but should not be used alone to mandate insulin initiation.	

## Introduction

Gestational diabetes mellitus is one of the most common medical complications of pregnancy and is associated with increased risks of hypertensive disorders, operative delivery, fetal overgrowth, neonatal metabolic complications, and future cardiometabolic disease in both mother and child [1, 3, 4–8]

Although insulin has historically been regarded as the standard pharmacologic treatment for gestational diabetes mellitus, metformin is increasingly used because of its ease of administration, lower maternal weight gain, lower risk of hypoglycemia, and high acceptability to patients [1–3]. Nevertheless, a substantial proportion of women treated with metformin ultimately require supplemental insulin, underscoring the importance of identifying predictors of metformin failure early in the course of care [1, 2, 15–18].

The diagnostic OGTT provides more than a binary diagnosis of GDM. Fasting hyperglycemia reflects impaired basal insulin secretion together with increased hepatic glucose output, whereas late post-load hyperglycemia may reflect delayed glucose clearance and greater peripheral insulin resistance [9–14]. These OGTT-derived patterns may therefore help identify distinct metabolic phenotypes with different responses to pharmacologic therapy.

The aim of this study was to evaluate whether impaired fasting glucose, an abnormal 180-min OGTT value, and other maternal clinical characteristics predict metformin failure in women with gestational diabetes mellitus.

## Materials and methods

### Study design and population

We conducted a retrospective cohort study of women with singleton pregnancies diagnosed with gestational diabetes mellitus at Meir Medical Center between 2018 and 2023.

Women with singleton pregnancies diagnosed with gestational diabetes mellitus who initiated metformin therapy were eligible for inclusion. Women with pregestational diabetes or multifetal gestations were excluded.

Gestational diabetes mellitus was diagnosed using the American Diabetes Association (ADA) two-step approach, consisting of an initial 50-g glucose challenge screening test followed, when abnormal, by a diagnostic 100-g, 3-h OGTT performed under fasting conditions and interpreted according to Carpenter-Coustan thresholds (fasting ≥ 95 mg/dL, 1-h ≥ 180 mg/dL, 2-h ≥ 155 mg/dL, and 3-h ≥ 140 mg/dL) [3, 5].

Routine screening for gestational diabetes mellitus was generally performed between 24 and 28 weeks’ gestation according to institutional and national guidelines, with earlier screening performed in high-risk patients when clinically indicated.

**Fig. 1:**
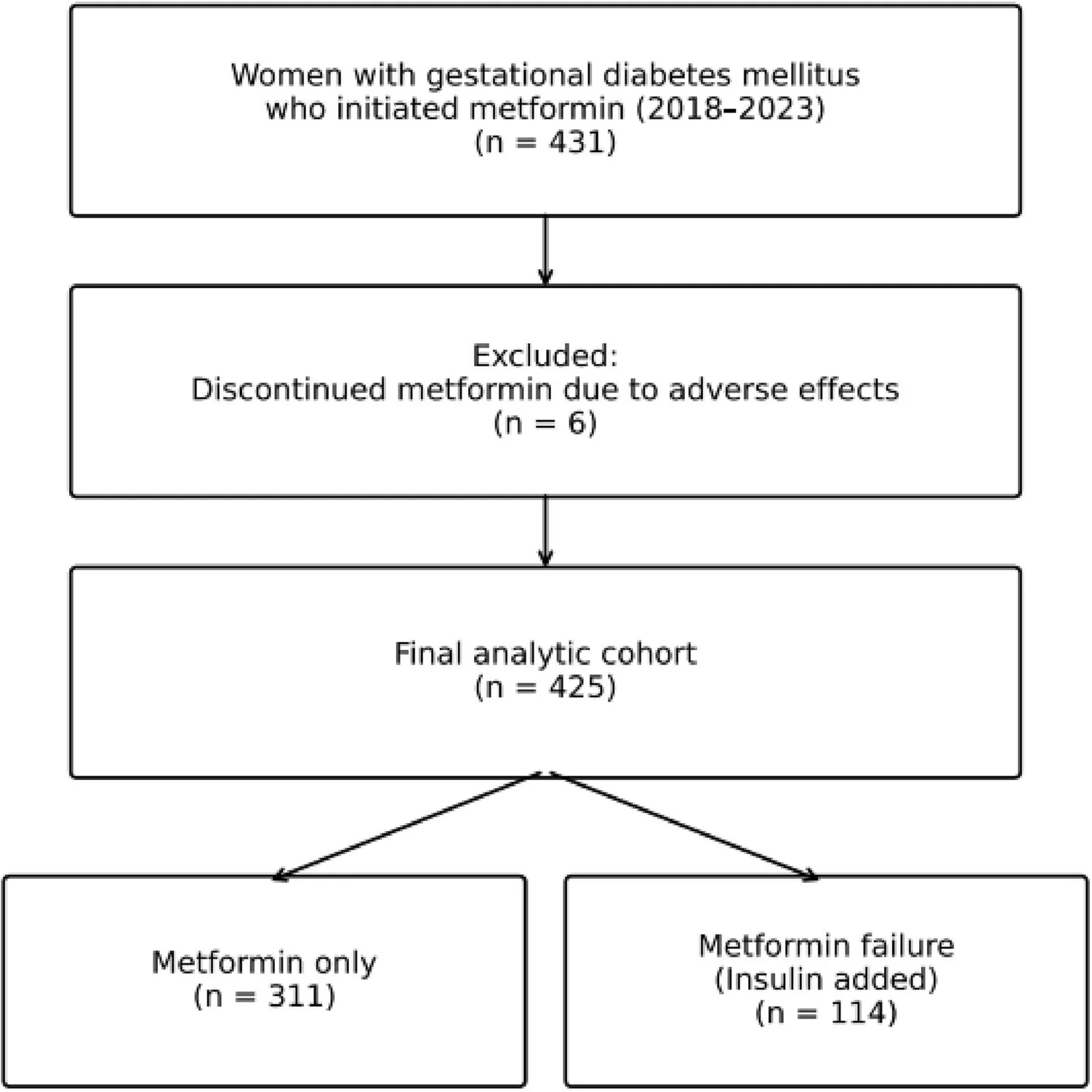
Flow diagram of cohort selection. Of 431 women with gestational diabetes mellitus who initiated metformin, 6 discontinued treatment due to adverse effects, leaving 425 women for analysis. Among them, 311 remained on metformin alone and 114 required insulin therapy

The source registry captured women who initiated metformin therapy after diagnosis of gestational diabetes mellitus. Of 431 women who initiated metformin, 6 discontinued therapy because of adverse effects and were excluded, yielding a final analytic cohort of 425 women. A cohort selection flow diagram is presented in Fig. 1.

### Medical nutrition therapy and pharmacologic escalation

All women received standard-of-care dietary counseling and self-monitoring of blood glucose as part of routine gestational diabetes management before or at the time of pharmacologic treatment escalation. Management of gestational diabetes in our institution follows standardized departmental protocols and is conducted by the obstetric team according to predefined clinical guidelines.

When pharmacologic therapy was indicated, metformin was initiated at a dose of 850 mg once daily. The dose was subsequently titrated according to glycemic control up to a maximum of three tablets daily (2550 mg/day), which represents the maximal dose used in our clinical practice.

Glycemic control was routinely reviewed by the treating physician based on patients’ self-monitored glucose measurements. Continuous glucose monitoring devices were not routinely used or systematically recorded in the study cohort during the study period, and glycemic management was therefore primarily based on self-monitored capillary glucose measurements.

In clinical practice, inadequate glycemic control was generally defined when more than 30% of recorded glucose values exceeded target thresholds. If glucose values remained above target despite treatment with metformin at the maximal tolerated dose (up to 2550 mg/day), insulin therapy was initiated and this situation was defined as metformin failure.

In routine clinical practice at our institution, women with persistent inadequate glycemic control despite maximal tolerated metformin therapy are generally escalated to insulin treatment according to standardized departmental protocols.

While these clinical data are available in the medical records, the retrospective structure of the registry did not allow uniform extraction of detailed individual glucose logs or adherence to medical nutrition therapy. Accordingly, these variables were not included as covariates in the statistical models.

### Definitions

The primary outcome was metformin failure, defined as subsequent initiation of insulin because glycemic control was considered inadequate on metformin monotherapy during routine clinical care.

Impaired fasting glucose was defined for this obstetric cohort as a fasting value ≥ 95 mg/dL on the diagnostic 100-g OGTT, corresponding to the Carpenter-Coustan fasting threshold used for gestational diabetes diagnosis [3]. An abnormal 180-min OGTT value was defined as a 3-h glucose value ≥ 140 mg/dL, again according to Carpenter-Coustan criteria [3].

### Predictors

Candidate predictors were selected a priori on the basis of physiologic plausibility and prior literature and included OGTT glucose values at fasting, 60 min, 120 min, and 180 min; IFG; abnormal 180-min OGTT value; number of abnormal OGTT values; prepregnancy BMI; gestational weight gain; maternal age; parity; previous neonatal birthweights; and gestational age at metformin initiation. The number of abnormal OGTT values was specifically evaluated as a potential predictor of metformin failure but was not independently associated with the outcome in multivariable analysis.

### Statistical analysis

Continuous variables are reported as mean (standard deviation) and categorical variables as number (percentage). Continuous variables were compared using Welch’s t test, and categorical variables were compared using the χ^2^ test or Fisher’s exact test, as appropriate.

Univariable logistic regression was used to examine the association between each predictor and metformin failure. Because fasting, 60-min, 120-min, and 180-min OGTT values are correlated measures of the same diagnostic test, the primary multivariable model retained prespecified categorical metabolic phenotypes rather than all continuous OGTT time-point values simultaneously in order to reduce multicollinearity and improve interpretability. Collinearity diagnostics were performed during model revision and did not demonstrate clinically significant multicollinearity in the final model.

The multivariable model included impaired fasting glucose, abnormal 180-min OGTT value, maternal age, prepregnancy BMI, gestational weight gain, parity, and gestational age at metformin initiation. Adjusted odds ratios with 95% confidence intervals were calculated.

## Results

Among the 425 women included in the analysis, 114 (26.8%) required supplemental insulin therapy, whereas 311 (73.2%) remained on metformin alone throughout pregnancy. Women who subsequently required insulin were slightly older and initiated metformin earlier in gestation. In contrast, prepregnancy BMI, gestational weight gain, parity, previous neonatal birthweights, and the number of abnormal OGTT values did not differ significantly between groups (Table 1).

**Table 1:** Baseline characteristics according to metformin failure

Characteristic	Total (n = 425)	Insulin (n = 114)	Metformin only (n = 311)	p value
Maternal age, years	33.7 (5.3)	34.6 (5.1)	33.4 (5.4)	0.038
Prepregnancy BMI, kg/m^2^	28.6 (6.5)	29.4 (5.0)	28.2 (7.1)	0.521
Gestational weight gain, kg	9.1 (10.8)	10.0 (7.5)	8.7 (11.7)	0.395
Gestational age at metformin initiation, weeks	29.8 (6.5)	27.9 (7.4)	30.5 (6.0)	0.004
Number of abnormal OGTT values	1.37 (1.13)	1.46 (1.49)	1.33 (0.97)	0.374
Previous largest birthweight, g	3475 (538)	3506 (633)	3465 (503)	0.641
Previous smallest birthweight, g	2999 (549)	2985 (590)	3004 (537)	0.845

Data are presented as mean (standard deviation) unless otherwise indicated

In unadjusted analyses, women who progressed to insulin therapy demonstrated significantly higher glucose concentrations at all OGTT time points, including fasting, 60 min, 120 min, and 180 min (all p ≤ 0.001). Impaired fasting glucose (IFG) was more prevalent among women who required insulin compared with those who remained on metformin alone (41.2% vs 26.4%, p = 0.005). Likewise, an abnormal 180-min OGTT value was substantially more common among women who progressed to insulin therapy (21.1% vs 5.1%, p < 0.001) (Table 2).

**Table 2:** OGTT profile and glycemic predictors according to metformin failure

Predictor	Total	Insulin	Metformin only	p value
Fasting glucose, mg/dL	98.1 (11.5)	102.7 (13.7)	96.8 (10.5)	0.001
60-min glucose, mg/dL	187.2 (32.5)	203.9 (33.4)	182.6 (30.7)	< 0.001
120-min glucose, mg/dL	148.2 (36.1)	163.8 (44.4)	143.8 (32.1)	< 0.001
180-min glucose, mg/dL	106.7 (32.0)	123.8 (39.3)	102.0 (28.0)	< 0.001
IFG, n (%)	129 (30.4)	47 (41.2)	82 (26.4)	0.005
Abnormal 180-min value, n (%)	40 (9.4)	24 (21.1)	16 (5.1)	< 0.001

IFG was defined as fasting OGTT value ≥ 95 mg/dL. An abnormal 180-min value was defined as ≥ 140 mg/dL

The number of abnormal OGTT values was evaluated as a candidate predictor but was not independently associated with metformin failure.

**Fig. 2:**
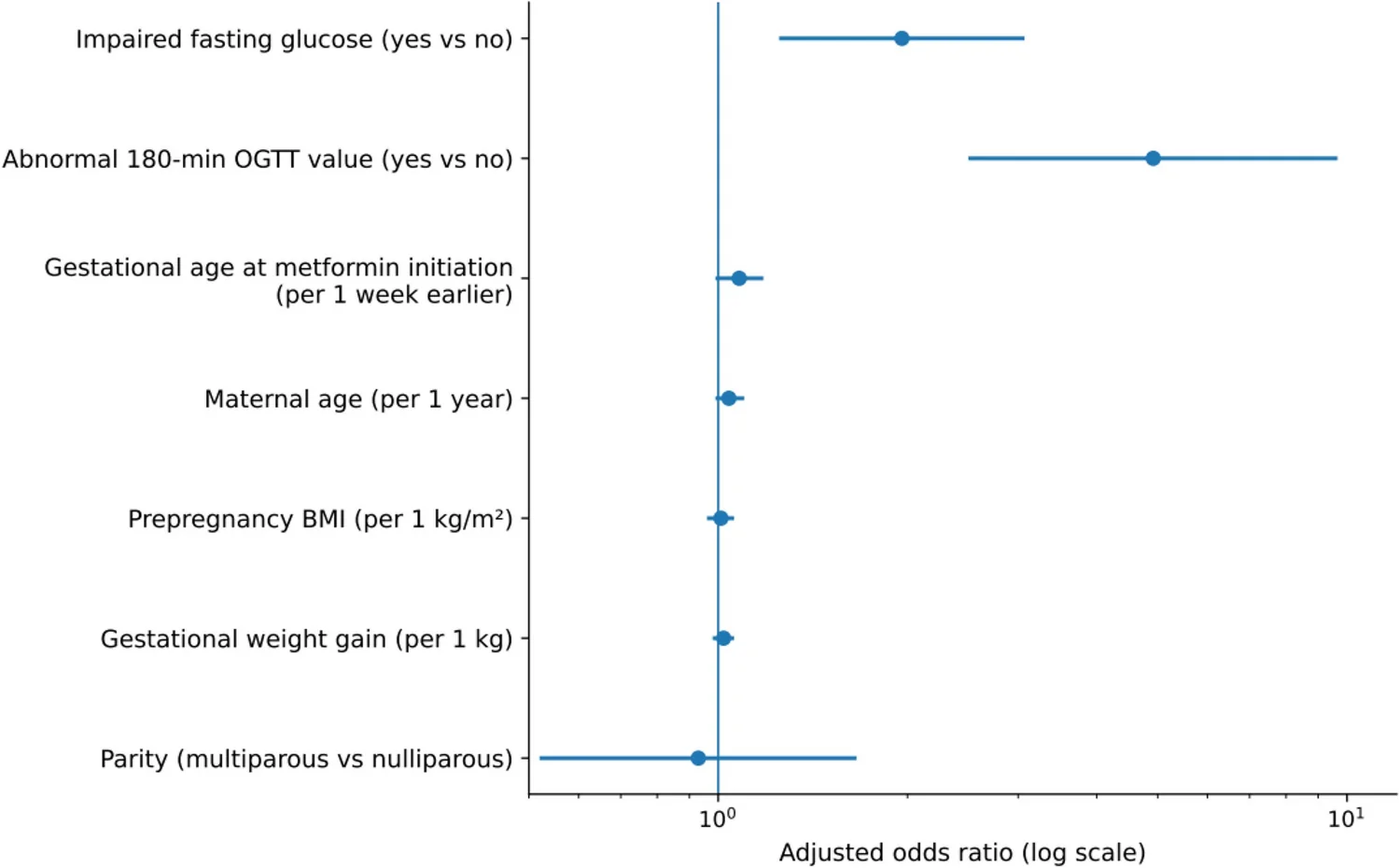
Predictors of metformin failure in gestational diabetes mellitus. Forest plot displaying adjusted odds ratios (aOR) with 95% confidence intervals derived from the primary multivariable logistic regression model. Impaired fasting glucose and an abnormal 180-min OGTT value were independently associated with increased odds of metformin failure. Other maternal and clinical variables, including gestational age at metformin initiation, maternal age, prepregnancy body mass index, gestational weight gain, and parity, were not significantly associated with the outcome

In the multivariable logistic regression model (Table 3 and Fig. 2), impaired fasting glucose and an abnormal 180-min OGTT value were independently associated with an increased likelihood of metformin failure (aOR 1.96, 95% CI 1.25–3.07, p = 0.005 and aOR 4.92, 95% CI 2.50–9.66, p = 0.003, respectively).

**Table 3:** Multivariable logistic regression model for metformin failure

Variable	Adjusted OR	95% CI	p value
Impaired fasting glucose (yes vs no)	1.96	1.25–3.07	0.005
Abnormal 180-min OGTT value (yes vs no)	4.92	2.50–9.66	0.003
Gestational age at metformin initiation (per 1 week earlier)	1.08	0.99–1.18	0.07
Maternal age (per 1 year)	1.04	0.99–1.10	0.11
Prepregnancy BMI (per 1 kg/m^2^)	1.01	0.96–1.06	0.74
Gestational weight gain (per 1 kg)	1.02	0.98–1.06	0.29
Parity (multiparous vs nulliparous)	0.93	0.52–1.66	0.80

The primary multivariable model retained prespecified categorical OGTT phenotypes rather than all continuous OGTT time-point values simultaneously because of collinearity among OGTT measures. Reference category for parity was nulliparity

Gestational age at metformin initiation showed a non-significant trend toward increased risk with earlier initiation (aOR 1.08, 95% CI 0.99–1.18, p = 0.07). Maternal age, prepregnancy body mass index, gestational weight gain, and parity were not significantly associated with metformin failure.

## Discussion

In this retrospective cohort study of women with gestational diabetes mellitus initially treated with metformin, approximately one quarter ultimately required supplemental insulin therapy. This rate is consistent with prior studies reporting that metformin monotherapy is insufficient for a clinically meaningful subset of women with GDM [1, 2, 15–18].

Our main finding was that impaired fasting glucose (IFG) and an abnormal 180-min OGTT value were independently associated with subsequent insulin requirement. These findings support the concept that OGTT-derived metabolic phenotypes may help identify women less likely to achieve adequate glycemic control with metformin alone.

Fasting hyperglycemia might be expected to respond to metformin because metformin primarily reduces hepatic gluconeogenesis. However, fasting hyperglycemia may reflect not only increased hepatic glucose output but also more severe underlying β-cell dysfunction, a greater overall glycemic burden, or an earlier stage of disease requiring pharmacologic intensification. Thus, IFG in this setting may function less as a marker of a specific mechanistic pathway of drug resistance and more as a clinical marker of greater disease severity. The observed associations likely reflect greater underlying metabolic severity rather than isolated effects of crossing the diagnostic thresholds themselves.

The association with an abnormal 180-min OGTT value is also biologically plausible. Late post-load hyperglycemia may reflect delayed glucose disposal and more pronounced peripheral insulin resistance, particularly in skeletal muscle [10–14]. The 180-min threshold of 140 mg/dL corresponds to the established Carpenter-Coustan diagnostic cutoff. While this threshold has not been universally validated as a specific marker of delayed glucose clearance, persistent late post-load hyperglycemia may physiologically reflect impaired peripheral glucose disposal and prolonged metabolic recovery after glucose challenge.

Importantly, these findings should not be interpreted as suggesting that women meeting Carpenter-Coustan diagnostic thresholds should automatically be considered candidates for insulin therapy. Rather, these OGTT-derived phenotypes may identify women at higher risk for subsequent treatment escalation and therefore warrant closer surveillance following metformin initiation.

### Clinical implications

The findings of this study have several potential clinical implications. The diagnostic OGTT is routinely performed as part of standard care for gestational diabetes and provides detailed metabolic information without additional testing or cost. Incorporating specific OGTT parameters—particularly fasting glucose and late post-load glucose levels—into clinical assessment may help identify women who are less likely to achieve adequate glycemic control with metformin monotherapy.

Early identification of such patients could enable closer glycemic surveillance after metformin initiation or earlier consideration of insulin therapy, potentially reducing fetal exposure to hyperglycemia and improving perinatal outcomes. More broadly, these findings support the concept that OGTT-derived metabolic phenotypes may contribute to a more individualized approach to pharmacologic management in GDM.

Early identification of such patients could enable closer glycemic surveillance after metformin initiation or earlier consideration of insulin therapy, potentially reducing fetal exposure to hyperglycemia and supporting more favorable fetal growth, including birthweight. More broadly, these findings support the concept that OGTT-derived metabolic phenotypes may contribute to a more individualized approach to pharmacologic management in GDM.

### Strengths and limitations

This study has several strengths. It is based on a well-characterized real-world cohort managed under a standardized departmental protocol, reflecting routine clinical practice. Predictors were selected a priori, and multivariable modeling was performed with adjustment for clinically relevant confounders. In addition, the analysis incorporated the full 3-h diagnostic OGTT profile rather than relying solely on fasting glucose values, allowing a more comprehensive evaluation of OGTT-derived metabolic patterns.

The study was conducted at a single center using a retrospective design. Detailed delivery and neonatal outcomes, including birthweight, were not consistently available in the analytic registry and were therefore not included in the present analysis.

The study was conducted at a single center using a retrospective design, and maternal and neonatal delivery outcomes—such as birthweight, macrosomia, and neonatal hypoglycemia—were not uniformly available in the analytic registry used for the present analysis and therefore were not incorporated into the current models.

Because of the retrospective design, detailed longitudinal data regarding the degree and duration of hyperglycemia before insulin escalation were not uniformly available in the registry.

In addition, adherence to medical nutrition therapy could not be uniformly quantified within the retrospective registry. Because nutritional therapy represents a cornerstone of gestational diabetes management, variability in adherence may have influenced the likelihood of subsequent insulin initiation and could therefore represent an unmeasured confounder.

## Conclusion

In women with gestational diabetes mellitus treated initially with metformin, IFG and an elevated 180-min OGTT value were associated with subsequent insulin requirement. These findings suggest that metabolic heterogeneity within gestational diabetes may influence response to metformin.

However, the results should be interpreted as hypothesis-generating and not as direct indications for early insulin initiation. Rather, these OGTT-derived phenotypes may help identify women who could benefit from closer glycemic surveillance and individualized management.

## Data Availability

No datasets were generated or analysed during the current study.
